# *P2RX7*: Expression Responds to Sleep Deprivation and Associates with Rapid Cycling in Bipolar Disorder Type 1

**DOI:** 10.1371/journal.pone.0043057

**Published:** 2012-08-28

**Authors:** Lena Backlund, Catharina Lavebratt, Louise Frisén, Pernilla Nikamo, Dzana Hukic Sudic, Lil Träskman-Bendz, Mikael Landén, Gunnar Edman, Marquis P. Vawter, Urban Ösby, Martin Schalling

**Affiliations:** 1 Department of Clinical Neuroscience, Karolinska Institutet, Stockholm, Sweden; 2 Neurogenetics Unit, Department of Molecular Medicine and Surgery, Karolinska Institutet, Stockholm, Sweden; 3 Center for Molecular Medicine, Karolinska University Hospital, Stockholm, Sweden; 4 Department of Clinical Sciences, University Hospital, Lund, Sweden; 5 Institution of Neuroscience and Physiology, The Sahlgrenska Academy at Gothenburg University, Sweden; 6 Functional Genomics Laboratory, Department of Psychiatry and Human Behavior, School of Medicine, University of California Irvine, Irvine, California, United States of America; 7 Department of Psychiatry, Tiohundra AB, Norrtälje, Sweden; University of Pennsylvania School of Medicine, United States of America

## Abstract

**Context:**

Rapid cycling is a severe form of bipolar disorder with an increased rate of episodes that is particularly treatment-responsive to chronotherapy and stable sleep-wake cycles. We hypothesized that the *P2RX7* gene would be affected by sleep deprivation and be implicated in rapid cycling.

**Objectives:**

To assess whether *P2RX7* expression is affected by total sleep deprivation and if variation in *P2RX7* is associated with rapid cycling in bipolar patients.

**Design:**

Gene expression analysis in peripheral blood mononuclear cells (PBMCs) from healthy volunteers and case-case and case-control SNP/haplotype association analyses in patients.

**Participants:**

Healthy volunteers at the sleep research center, University of California, Irvine Medical Center (UCIMC), USA (n = 8) and Swedish outpatients recruited from specialized psychiatric clinics for bipolar disorder, diagnosed with bipolar disorder type 1 (n = 569; rapid cycling: n = 121) and anonymous blood donor controls (n = 1,044).

**Results:**

*P2RX7* RNA levels were significantly increased during sleep deprivation in PBMCs from healthy volunteers (p = 2.3*10^−9^). The *P2RX7* rs2230912 _A allele was more common (OR = 2.2, p = 0.002) and the ACGTTT haplotype in *P2RX7* (rs1718119 to rs1621388) containing the protective rs2230912_G allele (OR = 0.45–0.49, p = 0.003–0.005) was less common, among rapid cycling cases compared to non-rapid cycling bipolar patients and blood donor controls.

**Conclusions:**

Sleep deprivation increased *P2RX7* expression in healthy persons and the putatively low-activity *P2RX7* rs2230912 allele A variant was associated with rapid cycling in bipolar disorder. This supports earlier findings of *P2RX7* associations to affective disorder and is in agreement with that particularly rapid cycling patients have a more vulnerable diurnal system.

## Introduction

Rapid cycling (RC) is a severe form of bipolar disorder, characterized by four or more disease episodes within one year (DSM-IV) [1], [2]. A RC course occurs in 12–24% of bipolar disorder patients [3]. In addition to more frequent episodes, RC implies greater functional impairment, increased risk of suicide attempts and a higher rate of alcohol abuse [1], [4]–[6]. Due to the clinical severity of RC, clinicians try to prevent its development by early active symptom treatment. In bipolar disorder, antidepressants are often necessary to treat bipolar depression, despite the possible risk for mood switches in vulnerable patients [7]–[10]. Methods to identify patients at risk for RC would be of great clinical value.

The heritability of bipolar disorder is estimated to 79–93% [11]. Several interesting candidate genes have been identified, but replication of these findings are scarce. This may relate to the heterogeneity of symptoms and course, comorbidity between bipolar disorder and other psychiatric disorders and genetic differences between ethnic groups and environmental effects. Specific symptoms and sub-phenotypes of bipolar disorder may represent different biological variants, and genetic association studies may be helpful to identify these. Some studies using this strategy have been promising, e.g. both persecutory delusions and an early onset of illness have been associated with the *DAOA* and *GRK3* genes [12]. Mood-incongruent psychotic symptoms have been linked to 1q32.3, 7p13 and 20q13.3 [13] and a favorable lithium-response to chromosome 10p15 [14]. Previously we have presented cognitive symptoms in mania associated with the *P2RX7* gene [15]. Case-case studies (comparing patients with specific symptoms to patients without such symptoms) may minimize the effect of environmental factors (i.e. stress, drug exposure, socio-economic factors) between patients [16]–[18]. We have used this strategy in a previous genetic study of RC in bipolar disorder [19]. Furthermore, a specific genetic RC vulnerability is suggested by a familial aggregation of RC [20], and associations to RC have been found in the *COMT* [21], *SLC6A4* [22], [23], *BDNF* [24], [25] and *CRY2* [19] genes.

Sleep disturbances and diurnal-related anxiety in bipolar episodes are associated with disturbed biological rhythm function [26]. Bipolar patients with RC have a more vulnerable diurnal system than those without RC [27]–[29]. Treatment studies also showing that both social rhythm therapy and psychotherapy focused on stable sleep-wake cycle is especially beneficial in RC bipolar patients [30], [31]. Most patients with RC have to bee treated with a combination of lithium and valproic acid. Lithium has been shown to stabilize the circadian rhythm in bipolar disorder [32]–[34]. In mice, lithium is proposed to prolong the circadian period in the suprachiasmatic nucleus (SCN) [35]. Valproic acid is also believed to influence the circadian rhythm, but through other mechanisms [36]. Altogether, this may explain the usefulness lithium and valproate in combination in RC maintenance treatment.

### The *P2RX7* gene

*P2RX7* is a candidate gene for bipolar disorder that was first identified by linkage analysis in a French-Canadian population [35]. The SNP rs2230912 was associated with bipolar disorder in a large and detailed family-based study [37]. Other polymorphisms in the *P2RX7* gene have been associated with mood and anxiety disorders [38]–[41]. The *P2RX7* gene has been suggested to be involved in the regulation of glutamate activation [42], [43] which has been proposed to to be involved in the circadian rhythm [44], [45]. Absence of P2rx7 in mouse brain have been shown to decrease depressive-like behavior and attenuate amphetamine-induced hyperactivity [46] whereas activation of the *P2RX7* gene may lead to glutamate over-activation and secondary to depressive symptoms [47].

We hypothesized that *P2RX7* expression in a healthy state is affected by disturbance in the circadian rhythm and further that polymorphisms in *P2RX7* are associated with RC which is a subtype of bipolar disorder associated with a more vulnerable diurnal rhythm.

## Methods

### Ethics statement

In the expression study informed consent was obtained from each participant using an approved University of California Institutional Review Board (IRB) protocol. All these participants were healthy individuals. The genetic study was approved by the Regional Ethical Review Board in Stockholm in accordance with the Helsinki Declaration of 1975. All bipolar participants were in euthymic phase. In both studies all individuals had full capacity to consent and the informed consent process was both verbal and written during a visit to a special trained psychiatric nurse.

### Participants in the RNA expression study

**Figure 1:**
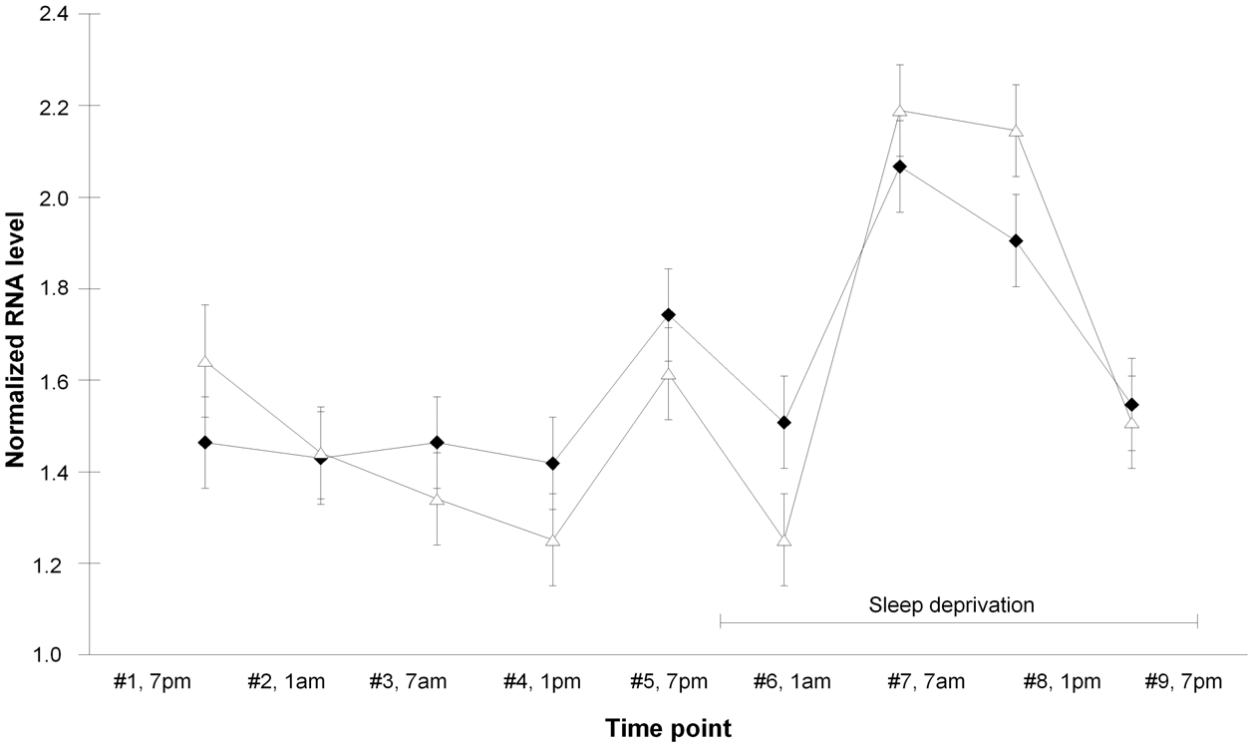
*P2RX7* gene expression in peripheral blood mononuclear cells from healthy volunteers. *P2RX7* RNA expression was increased by sleep deprivation in men (filled diamonds) and women (open triangles). Blood draw (x-axis) was at 7 p.m. and thereafter once every 6 hours. Sleep deprivation was at time points #6, #7, #8 and #9. RNA levels are the normalized average of exon expression across the entire RefSeq transcript. Diamonds and triangles indicate mean and error bars indicate SEM.

Eight healthy volunteers (4 women, 4 men, all with European descent), aged 24 years on average (SD = 5.9, range = 19–34 years), were investigated at the sleep research center at University of California at Irvine Medical Center (UCIMC) for 48 hours and deprived of sleep for 36 hours after an overnight stay (Figure 1). During sleep deprivation, wakefulness was maintained by allowing activities such as walking, reading, watching television, and playing card games. Subjects were not permitted to consume caffeinated foods or beverages to stay awake. Subjects' wakefulness was ensured by research assistants. Venous blood samples were drawn at 9 different times, beginning at 7 p.m. and every 6 hours thereafter. The blood was collected standard acid citrate dextrose (ACD) tubes (Becton Dickinson, Franklin Lakes, NJ, USA). Within 60 minutes in room temperature (RT) after blood drawl, the whole blood samples were centrifuged at 1500 rpm for 10 min at RT and the upper layer was thereafter transferred onto Ficoll-Paque (Amersham Biosciences, Piscataway, NJ, USA). Peripheral blood mononuclear cells (PBMC) were separated by density gradient centrifugation at 2500 rpm at RT for 20 min. The resulting ‘buffy’ coat layer was added to 10 ml phosphate buffered saline (PBS) at pH of 7.4 and centrifuged at 1000 rpm for 10 min at RT. The resulting pellet (5−10×10^6^ cells) was resuspended in 1 ml Trizol and total RNA was extracted using the standard Trizol isolation protocol (Invitrogen, Carlsbad, CA, USA). The RNA was resuspended in 100 µL diethyl pyrocarbonate (DEPC) treated water, analyzed for quality and quantity on a 2100 Bioanalyzer (Agilent, Palo Alto, CA, USA) and concentration was adjusted to 1 µg/µl.

RNA expression levels were determined using the Affymetrix oligonucleotide microarray chip (Human Gene- Chip Exon 1.0 ST) and the expression profiling experiments were carried out following the manufacturer's technical protocol (Affymetrix, Santa Clara, CA). In brief, total RNA was first subjected to a ribosomal RNA removal procedure (RiboMinus Human/Mouse Transcriptome Isolation Kit, Invitrogen) to reduce the 28S and 18S rRNA population significantly minimizing background and increasing sensitivity. Reduced RNA was reverse-transcribed to cDNA with random hexamers tagged with a T7 promoter sequence followed by second strand cDNA synthesis using DNA polymerase. The double stranded cDNA was then used for amplification of antisense cRNA and cleaned (GeneChip Sample Cleanup Module). A second cycle cDNA synthesis reaction was performed using random primers to reverse transcribe the cRNA into sense single stranded DNA which was then fragmented, and hybridized to the Affymetrix Human Genome Exon Arrays. Arrays were washed, stained, and scanned on the Affymetrix Fluidics Station and G7 Affymetrix high-resolution scanner using GCOS 1.3. The CEL files derived from the 1.0 ST chips were analyzed with a robust multi-array condensation algorithm (RMA) [48]. All the expression data for *P2RX7* is listed in Table S1 in a MIAME compliant format.

The quality control of each exon array was evaluated by using ExACT (Affymetrix software) for all CEL files. Based upon meeting Affymetrix quality control standard and principal component analysis, all 71 exon arrays were included in the analysis, that is, 9 samples per healthy volunteer collected day 1 at 7 p.m., day 2 at 1 am, 7 a.m., 1 p.m. and 7 p.m., as well as day 3 at 1 a.m., 7 a.m., 1 p.m. and 7 p.m. where the volunteers were deprived from sleep after the day 1 to day 2 night. Time point day 1 at 7 p.m. did not have high quality RNA from one of the healthy volunteers and was therefore not run on an exon array. Each array was individually normalized using an individual mean center. Data were analyzed in Partek Genomic Solutions (St. Louis, MO) and a total of 1,389,155 probes were imported into Partek after filtering out probes with SNPs. The RNA levels reported are the average of exon expression (24 probe sets) across the entire RefSeq transcript (transcript ID 3434726).

### Real-time quantitative PCR (qPCR) for validation

cDNA was generated using TaqMan reverse transcription (RT) reagents (Applied Biosystems). Each 50 µl RT reaction mix contained 5 µl of 10× Taqman RT buffer; 11 µl of 25 mM MgCl_2_; 10 µl of deoxy NTPs; 2.5 µl of Oligo d(T)_16_ primer; 1 µl of RNase inhibitor; 1.25 µl of Multiscribe reverse transcriptase, and 1 µl of RNA (1 µg/µl). The reaction mixtures were run on a GeneAmp PCR System 9700 (Applied Biosystems). The thermal cycling conditions were as follows: 25°C for 10 min for primer incubation, 48°C for 30 min for reverse transcription, and 95°C for 5 min for inactivation of reverse transcriptase. All cDNAs were stored at −20°C. qPCR was performed for *P2RX7* exon 3 (near the hybridization site for the probe (Probe Set 2950343; Affymetrix, Inc.)), and the housekeeping genes *GAPDH* and *TFG*. Primer sequences were as follows: *P2RX7* forward primer: 5′-AGGAAGAAGTGCGAGTCCAT-3′, reverse primer: 5′-CTGCTGGTTCACCATCCTAA-3′; *GAPDH* forward: 5′-ACCCACTCCTCCACCTTTGA-3′, reverse: 5′-AATTCGTTGTCATACCAGGA-3′; and *TFG* forward: 5′-TCGTTCAGACTGAGATCATTTAGACA-3′, reverse: 5′-TTTTCTCTGGGCACTTCAATTTC-3′. The primers were tested by using a set of brain cDNA samples (2 individual cDNAs from cerebellum), genomic DNA, no temple control (NTC), and RT minus (2 individual DLPFC RNA without cDNA) in duplicate and run on an ABI 7900HT Sequence Detection System (Applied Biosystems). The primer test results showed that all cDNA amplified well, the NTC and gDNA had a cycle threshold (Ct) greater than 40 and RT minus was 100-fold less than cDNA concentration. This detection ensured that the primers were not measuring residual genomic DNA, and the melting and amplification curves did not show any contribution of primer-dimers to the measurements.

The qPCR was performed on the ABI 7900HT in 384-well plates. The samples were assembled by Robot Biomek3000 (Beckman Coulter) and run with triplicates in one plate per gene. The reaction was performed in a 12.5 µl total volume with 6.25 µl of 2× Power SybrGreen master mix (Life Technologies); 0.25 µl of 10 µM forward primer; 0.25 µl of 10 µM reverse primer; 2 µl of a 1∶10 dilution of cDNA template (corresponding to approximately 4 ng RNA), and water to a total volume of 12.5 µl. The thermal cycling profiles were as follows: pre-steps at 50°C for 2 min (incubation) and 95°C for 10 min (activation), followed by 45 cycles at 95°C for 15 sec (denaturation) and 60°C for 1 min (annealing/extension), then by a dissociation step at 95°C for 15 sec, 60°C for 15 sec, and 95°C for 15 sec.

The cycle threshold (Ct) was determined approximately in the middle of the exponential phase of the amplification. The average and standard deviation (SD) and CV of triplicate Ct were calculated and the average value was accepted if the SD was lower than 0.39. A variation of the GENorm quantification method [49] was used to normalize gene expression. *GAPDH* and *TFG* were the selected housekeeping genes based upon in-house data. Each gene was individually scaled to the lowest Ct, and after scaling, the mean of the two housekeeping genes was used to normalize *P2RX7*.

### Participants in the genetic study

Patients (n = 646) at clinical diagnosis of bipolar disorder type 1 were recruited in Sweden, most of them from specialized outpatient clinics for affective disorders (n = 582; the Huddinge cohort: n = 509, the S:t Göran cohort: n = 73) and some from ordinary psychiatric outpatient clinics (n = 64). Life-time manic and depressive symptoms were assessed. The bipolar diagnosis was validated by noting the following manic symptoms: elevated mood, irritability, over-activity, grandiosity, decreased sleep, talkativeness, distractibility, goal-directed behavior, thought disorder, and embarrassing behavior. The assessment was based on interviews and medical records focusing on the most severe manic episode and performed by a psychiatrist specialized in bipolar disorder or by a trained psychiatric nurse. Manic and depressive symptoms were assessed using the modules for mania and depression in the Schedules for Clinical Assessment in Neuropsychiatry (SCAN; Table 1) [50], or with the Affective Disorder Evaluation as has been described elsewhere [51]. Depressive symptoms were assessed according to DSM-IV. On the basis of these assessment patients were considered as fulfilling the diagnostic criteria for bipolar disorder type 1, 2, or not otherwise specified (NOS). The phenotypes RC, mixed episodes and the age of onset of mania as well as of depression were also assessed. Patients who did not fulfill the DSM-IV criteria for bipolar disorder type 1 were excluded (n = 59). According to the DSM-IV definition of bipolar disorder, individuals were excluded if a manic episode was a result of alcohol or drug abuse, medication or somatic disease (n = 7). Also excluded were patients with close relatives already included in the study (n = 11). Another nine patients withdrew from the study. A total of 569 bipolar type 1 patients (42% men) were included in the study. Within the final study group 508 (88%) patients had been hospitalized for an affective episode at least once, 172 (30%) had mixed episodes and 121 (21%) were diagnosed as suffering from RC. Anonymous ABD controls (ABD; n = 1044, 59% men) recruited from Karolinska University Hospital, Stockholm, Sweden, were used as controls. They were between 18–70 years and not allowed to be on sick leave. They were negative for hepatitis B, C, HIV and syphilis, and requested to wait up to 6 months after being exposed for a risk for blood borne infection such as major surgical intervention, blood transfusion, accidental pin-prick, tattoo, piercing, sex with a new partner, or visit to a malaria endemic country. Furthermore, intravenous user of illicit drugs and men who had had sex with men were not allowed to donate blood.

**Table 1:** Clinical characteristics of the sample set that are genotyped (n = 569).

Bipolar disorder type 1	569
Men [n (%)]	241 (42)
Women [n (%)]	328 (58)
Age at first depression (md, range)	23.5 (4–64)
Age at first mania (md, range)	29.0 (6–70)
Hospitalized for affected episodes [n (%)]	508 (88)
Mixed episodes [n (%)]	172 (30)
Rapid cycling [n (%)]	121 (21)
Non-rapid cycling [n (%)]	446 (78)

### Genotyping

Peripheral blood samples were drawn and genomic DNA was extracted by standard procedures. Single nucleotide polymorphisms (SNPs) were selected from the HapMap database (www.hapmap.org). All SNPs were genotyped on a 7900HT Fast Real-Time PCR System Instrument using allele-specific Taqman MGB probes labeled with fluorescent dyes FAM and VIC (Applied Biosystems), according to the manufacturer's instructions. Allelic discrimination was performed with the ABI PRISM 7900HT SDS and the SDS 2.2.1 program (Applied Biosystems). Ten percent of the samples were run as duplicates to check for genotyping errors.

*P2RX7* genetic variants with a putative functionality were studied. Eight non-synonymous SNPs in the *P2RX7* gene were selected: rs208294 (His155Tyr), rs7958311 (Arg270His), rs28360457 (Arg307Gln), rs1718119 (Thr348Ala), rs2230911 (Thr357Ser), rs2230912 (Gln460Arg), rs3751143 (Glu496Ala) and rs1653624 (Ile568Asn) as well as one synonymous SNP: rs1621388 and finally one essential splice site SNP rs35933842 (Table 2).

**Table 2:** SNPs analyzed in *P2RX7* gene.

			MAF	MAF	MAF	Genotyping
SNP	Region^a^	Variation^b^	RC	nonRC	controls	success rate(%)
rs35933842 (A/C*)	Intron 1	Essential Splice site	0.0045	0.0056	0.0053	87.5
rs208294 (A/G*)	Exon 5	His155Tyr	0.39	0.45	0.42	86.4
rs7958311 (A/G*)	Exon 8	Arg270His	0.25	0.28	0.26	95.8
rs28360457 (A/G*)	Exon 9	Arg307Gln	0.023	0.015	0.012	87.2
rs1718119 (A/G*)	Exon 11	Ala348Thr	0.36	0.39	0.40	93.6
rs2230911 (G/C*)	Exon 11	Thr357Ser	0.11	0.069	0.097	92.5
rs2230912 (G/A*)	Exon 13	Gln460Arg	0.076	0.15	0.16	99.0
rs3751143 (C/A*)	Exon 13	Glu496Ala	0.16	0.16	0.15	92.7
rs1653624 (A/T*)	Exon 13	Ile568Asn	0.046	0.033	0.030	92.8
rs1621388 (A/G*)	Exon 13	Synonymous	0.37	0.40	0.40	96.8

a Data from www.ensembl.org . Minor allele first.

b Minor allele first, data from www.hapmap.org.

* Ancestral allele in CEU population data (CEPH (Utah residents with ancestry from northern and western Europe)) from www.ncbi.nlm.nih.gov.

MAF, minor allele frequency.

### Statistical analysis

For the array data, differential gene expression between time points was analyzed by repeated measures ANOVA applying a Huynh and Feldt-correction with sex and time point * sex interaction as fixed effects and time-point as a within-subject effect. For validation of differential expression of *P2RX7* by sleep deprivation, a similar ANOVA model was applied on qPCR data where time point was before (denoted 0, i.e. expression level being the mean of time points #1 to #5) or during sleep deprivation (denoted 1, i.e. time points #6 to #9). P<0.05 was regarded as significant in the qPCR validation analysis.

The genetic association between P2RX7 and bipolar disorder type 1 patients with RC versus non-RC bipolar disorder type 1 patients (nonRC) was first investigated in a case-case model. Second, the association was tested between RC and population-based controls (ABD) in a case-control analysis [16]. In both the case-case and the case-control analyses, the allele frequency difference was tested for the ten *P2RX7* SNPs using logistic regression. Since RC has been reported to be more common in women, gender was used as covariate [1]. The Hardy-Weinberg equilibrium (HWE) was evaluated for each SNP using a χ^2^-test. The measure D′ of linkage disequilibrium (LD) was calculated between the SNPs using the ABD controls, and haplotype blocks were constructed according to criteria proposed by Gabriel et al., 2002 [52]. Haplotype distribution for the RC compared to the nonRC patients and ABD controls was analysed using the χ^2^-test whereas the associations of a specific haplotype to case were calculated using logistic regression with gender as covariate. P-values reported in the genetic analyses are uncorrected for multiple testing. The Bonferroni corrected threshold considering the partial LD between markers p<0.017 (0.017 = 0.050/3 (3 SNP groups (defined by D′>0.80))) was regarded as significant in the allelic association tests, whereas p<0.05/6 = 0.0083 (6 haplotypes) was regarded significant in the analyses of specific haplotypes [53], [54]. The power was >0.75 to detect an association between RC and *rs2230912* for an allelic, a dominant and a co-dominant model in the case-case design whereas it was >0.82 in the case-control design. The corresponding power for recessive model was 0.41 for both designs. The power to detect RC association to the other SNPs was<0.35 (http://pngu.mgh.harvard.edu/purcell/gpc/).

## Results

### *P2RX7* and sleep deprivation

Using array data *P2RX7* RNA levels in PBMCs were significantly increased by total sleep deprivation in healthy volunteers (p = 2.3*10^−9^). Sex did not influence the *P2RX7* mRNA levels (p>0.85). That similar finding was found for both male and female samples constituted a replication since male and female samples were collected at different occasions (Figure 1). This was validated using qPCR showing a significant increase of *P2RX7* RNA levels from before sleep deprivation (time points #1 to #5) to during sleep deprivation (time points #6 to #9) (F = 7.1, df = 1, P = 0.037, partial η^2^ = 0.54) where sex and sex*time point interaction had no effect (p>0.15). The individual qPCR-based increase in *P2RX7* RNA level by sleep deprivation was for the male samples 119%, 592%, 45% and 151%, and for the female samples −26%, −7%, 20% and 231%.

### *P2RX7* and rapid cycling

**Figure 2:**
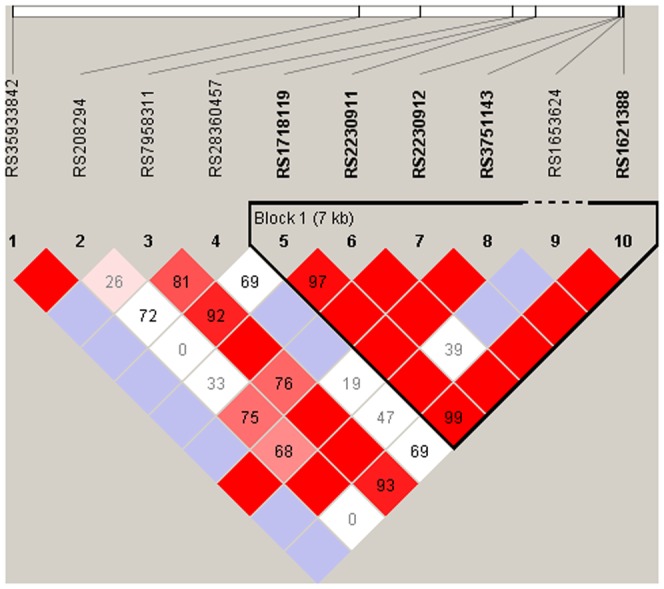
The strength of linkage disequilibrium (LD) between pairs of SNPs in ABD controls for *P2RX7*. The heavy-line frame shows suggested haplotype blocks. The numbers in the squares represent the pair-wise D′ value, empty squares stand for D′ = 1. Pink-red color indicates a pair-wise LOD≥2 with redness proportional to D′. White-blue square indicates LOD<2.

The major allele rs2230912_A, was more common among RC patients than among nonRC patients (OR = 2.2, p = 0.0027, 95% CI = 1.3–3.6; corresponding to OR = 0.45 for allele G; Table 3). Similarly, the allele rs2230912_A was more frequent among RC patients than among ABD controls (OR = 2.2, p = 0.0016, 95% CI = 1.4–3.7, corresponding to OR = 0.45 for rs2230912_G; Table 3). Linkage disequilibrium analysis of the ten *P2RX7* genotyped SNPs showed that six of them (from rs1718119 to rs1621388) formed an LD block (Figure 2). A haplotype association analysis was performed comparing RC with nonRC patients. Six haplotypes of the *P2RX7* block were identified and a difference in distribution of haplotypes between the RC and nonRC patients was found (χ^2^ = 13.0, df = 5, p = 0.023). The risk allele rs2230912_A was present in five haplotypes, whereas the rs2230912_G allele was present in only one haplotype (haplotype 1). An analysis of which specific haplotypes that differed in frequency between the RC and nonRC patients was performed. Consequently, the haplotype 1: ACGTTT (rs1718119 to rs1621388), including the protective G-allele from rs2230912, was less common in RC than in nonRC patients (OR = 0.45, p = 0.0031, 95% CI = 0.3–0.8; Table 4). As expected, this haplotype association was supported by the comparison between RC and ABD controls. This comparison showed that the ACGTTT haplotype was less common in RC patients compared to ABD controls (OR = 0.49, p = 0.0050, 95% CI = 0.3–0.8), and that there was an almost significant difference comparing the distribution of all haplotypes between RC and ABD controls (χ^2^ = 11.0, df = 5, p = 0.052; Table 4) .

**Table 3:** Allelic associations for RC compared to nonRC bipolar patients and for RC compared to ABD controls in the *P2RX7* gene.

	Bipolar disorder type 1							Blood donors as controls				
	RC		nonRC					Controls				
SNP^a^	aa/ab/bb	n	aa/ab/bb	n	p^b^	EMP1^c^	OR [95% CI]*	aa/ab/bb	n	p^b^	EMP1^c^	OR [95% CI]*
rs35933842 (A/C)	0/1/110	111	0/4/352	356	0.87	0.50	0.83 [0.089–7.2]	0/10/932	942	0.89	0.70	0.86 [0.11–6.7]
rs208294 (A/G)	17/52/42	111	70/173/107	350	0.12	0.11	0.78 [0.59–1.1]	158/463/310	931	0.51	0.43	0.91 [0.66–1.2]
rs7958311 (A/G)	8/39/63	110	34/151/209	394	0.44	0.55	0.88 [0.61–1.2]	69/409/562	1040	0.62	0.75	0.92 [0.68–1.3]
rs28360457 (A/G)	0/5/105	110	0/11/345	356	0.45	0.86	1.5 [0.51–4.3]	0/22/917	939	0.12	0.087	2.2 [0.74–5.2]
rs1718119 (A/G)	17/52/51	120	70/208/166	444	0.37	0.56	0.87 [0.81–1.4]	161/433/350	944	0.29	0.28	0.86 [0.79–1.3]
rs2230911 (G/C)	1/24/94	119	3/53/372	428	0.051	0.043	1.6 [1.0–2.7]	8/166/769	943	0.44	0.48	1.2 [0.74–1.8]
rs2230912 (G/A)	0/18/101	119	7/119/315	441	0.0027	0.0026	0.45 [0.28–0.77]	26/274/735	1035	0.0016	0.0015	0.45 [0.27–0.72]
rs3751143 (C/A)	2/34/83	119	12/110/304	426	0.93	0.86	1.0 [0.69–1.5]	20/240/686	946	0.55	0.59	1.1 [0.76–1.6]
rs1653624 (A/T)	0/11/108	119	0/28/402	430	0.34	0.44	1.4 [0.71–2.9]	2/52/892	946	0.21	0.17	1.5 [0.82–3.1]
rs1621388 (A/G)	17/49/47	113	69/205/152	426	0.35	0.56	0.86 [0.64–1.2]	174/471/375	1020	0.37	0.33	0.88 [0.65–1.2]

a SNP name (minor allele/major allele).

b gender as covariate, not corrected for multiple testing.

c Point-wise p-value from 10,000 permutations with no covariate (EMP1).

* Odds ratio (OR), the proportion of minor versus major allele among affected (RC)/proportion of minor versus major allele among unaffected (nonRC or controls).

**Table 4:** Haplotype association for RC compared with nonRC patients and RC patients compared with blood donor controls, in the *P2RX7* gene.

RC compared with nonRC									
*P2RX7*	rs1718119	rs2230911	rs2230912	rs3751143	rs1653624	rs1621388	F_RC_/F_nonRC_	p^a^	OR [95% CI]^b^
Haplotype 1	A	C	G	T	T	T	8.2/16.1	0.0029	0.45 [0.28–0.78]
Haplotype 2	A	C	A	T	T	T	28.2/24.6	0.28	1.2 [0.87–1.7]
Haplotype 3	G	C	A	T	A	C	5.0/3.0	0.14	1.8 [0.83–3.5]
Haplotype 4	G	C	A	G	T	C	16.4/15.2	0.68	1.1 [0.73–1.6]
Haplotype 5	G	G	A	T	T	C	10.0/6.9	0.13	1.5 [0.90–2.5]
Haplotype 6	G	C	A	T	T	C	32.3/34.3	0.58	0.91 [0.67–1.3]

RC compared with controls									
Haplotype 1	A	C	G	T	T	T	8.2/15.4	0.0050	0.49 [0.30–0.81]
Haplotype 2	A	C	A	T	T	T	28.2/24.3	0.23	1.2 [0.91–1.7]
Haplotype 3	G	C	A	T	A	C	5.0/3.0	0.12	1.7 [0.88–3.3]
Haplotype 4	G	C	A	G	T	C	16.4/14.9	0.57	1.1 [0.77–1.6]
Haplotype 5	G	G	A	T	T	C	10.0/9.7	0.88	1.0 [0.65–1.6]
Haplotype 6	G	C	A	T	T	C	32.3/32.6	0.92	0.99 [0.73–1.3]

F_cases_/F_controls_, percent with that haplotype in the RC group/non-RC/ABD controls with that haplotype, with successful haplotyping.

a not corrected for multiple testing.

b Odds ratio (OR), the ratio specific haplotype versus all other haplotypes among RC patients/ratio specific haplotype versus all other haplotypes among nonRC patients.

## Comments

The findings of this study were that *P2RX7* expression was affected by sleep deprivation in healthy volunteers and that a functional polymorphism in the *P2RX7* gene was associated to rapid cycling (RC) in bipolar type 1 patients in comparison to other bipolar disorder type 1 patients and anonymous blood donors. The study of *P2RX7* expression was hypothesis-driven based on (i) previously reported *P2RX7* genetic associations to bipolar disorder [39,39], and (ii) circadian rhythm disturbances reported in these patients [26] and (iii) previous findings of mood dysregulation in mice lacking *P2RX7* expression in the brain [46]. The here reported 1.5-fold increase of *P2RX7* expression (mean level of 24 probe sets over the entire RefSeq transcript) by sleep deprivation is only slightly lower than the previously reported 2.0 fold increase of *CRY2* expression level by sleep deprivation found in the same healthy volunteers and validated by real-time PCR [55]. *CRY2* participates in the core circadian loop. No difference in effect of sleep deprivation on *P2RX7* expression level was found between males and females, ensuring robustness in the analysis since males and females were investigated at different occasions. Since bipolar disorder patients with RC have a more vulnerable diurnal system than those without RC [27]–[29] and psychotherapy focused on stable sleep-wake cycle is especially beneficial in RC bipolar patients [30], [31], RC patients were investigated in the genetic study. RC was associated with a major allele of *P2RX7* (Gln460) previously reported to have lower activity than the alternative variant (Arg460). Thus, the association between RC and a *P2RX7* genetic variation implicated in *P2RX7* activity, and the sleep deprivation-induced increase of *P2RX7* expression level – sleep deprivation influences circadian rhythm - can be considered to be in agreement with the clinically well-established knowledge that RC patients have a more vulnerable diurnal system than those without RC. A lower *P2RX7* activity in RC may result in a less appropriate *P2RX7* response to sleep deprivation which may in part explain a vulnerable diurnal system and consequently more frequent episodes.

All the patients participating in this study of RC were patients from clinics with catchment responsibility for all patients with bipolar 1 disorder in the catchment area. Therefore, the participants may represent patients treated for bipolar 1 disorder in the general population of the catchment area. Almost all patients were recruited from specialized affective disorder units, all the medical records were studied by two investigators and most of the patients were also interviewed, resulting in a thorough phenotype assessment process. Cases as well as controls were ascertained from an ethnically homogeneous population [56] and further, controls were recruited from the same catchment area as the patients, thus reducing bias due to the ethnic variation. The prevalence of RC in bipolar disorder varies (12–24%) [3], which may result from different diagnostic procedures and/or differences between populations. Patients diagnosed as nonRC might develop RC after the assessment but nearly 30 percent of bipolar patients appear with RC in the early course of the disease and the increased rate of episodes would therefore be more likely in the first years of illness [57]. This indicates that probably only a limited number of the patients may change status to a RC course later.

The *P2RX7* gene is highly polymorphic and encodes the purinergic receptor P2X7 present in microglia, astrocytes and neurons in several brain regions [58]. This receptor is a ligand-gated calcium-permeable cat ion channel activated by ATP, which is involved in Ca^2+^-dependent signaling pathways [42]. The expression of *P2RX7* is high especially in the sub thalamic nucleus, hypothalamus and substantia nigra, all structures known to be associated with bipolar disorder (https://www.nextbio.com/b/search/ba/p2rx7?type=feature&id=19358).There is strong evidence that *P2RX7* promotes excitatory neurotransmitter release at presynaptic sites from neurons [43]. The SNP rs2230912 polymorphism in *P2RX7* results in a glutamine–to–arginine change (Gln460Arg), which is likely to affect *P2RX7* dimerization and protein–protein interactions [59]. The Arg-variant (allele G) resulted in enhanced P2X7 pore activity in human monocytes [60]. The gene is located on chromosome 12q24 at the center of a strong bipolar disorder linkage peak [37]. *P2RX7* has previously been associated with bipolar disorder, depression, anxiety disorders [38], [40], [41], [61], and cognitive symptoms in mania [15] and is believed to play a role in antidepressant action and causation of bipolar disorder by influencing neurotransmission [62], neuroprotection [63], and neuroinflammatory responses [64]. In agreement with our finding that the relatively rare RC phenotype had a higher frequency of the allele rs2230912_A compared to the rest of the bipolar disorder type 1 patients, previous studies have shown that bipolar disorder, depression and anxiety disorders were associated with the rs2230912_G allele [40], [41]. However, frequency of rs2230912_G was similar in nonRC and anonymous ABD controls in this study.

*P2RX7* may be involved in the diurnal rhythm regulation through indirect regulation of glutamate levels. Activated P2X7 channels have been proposed to mediate release of cytosolic glutamate [65]. In general, glutamate concentration increases rapidly and progressively during wakefulness and REM sleep, and decreases during non-REM sleep. Levels of glutamate receptors are altered between sleep and waking periods to keep the concentration of extracellular glutamate within a homeostatic range across sleep–waking states [66], [67]. However, alternatively the increased *P2RX7* expression seen upon sleep deprivation might be a stress response to the sleep deprivation.

Bipolar disorder and unipolar depression patients often show a disturbed phase in their circadian rhythm. Further, sleep deprivation is an effective short-term treatment for depression. The biological basis of the anti-depressive effect of sleep deprivation is not clear, but there is evidence that sleep deprivation resets the circadian rhythm. The P2RX7 through P2X7 receptors induces a higher permeability for calcium, leading to an increased intracellular calcium level and activated cytokines in the limbic dopaminergic pathways [68], [69]. We found the putatively low-activity P2RX7 rs2230912 allele A variant to be associated with RC in bipolar disorder which supports earlier findings of P2RX7 associations to affective disorder [37], [39], [40], [59]. We found no association between RC and anyone of the other SNPs analyzed. However, due to lack of power true single SNP association to RC for any of the SNPs without detected association cannot be excluded. The findings may lead to a better understanding of the biology behind RC in bipolar disorder but further studies are needed for validation. The result also illustrates the potential of studying defined subtypes of bipolar disorder and of applying a case-case design.

## Supporting Information

Table S1 **Expression data for** ***P2RX7*** **listed in a MIAME compliant format.** Raw and normalized data for each 71 samples ×24 probesets covering 18 exons is listed. The 71 samples consist of 8 individuals (subjects) sampled at 9 time points.
(XLS)
